# Nitrous Oxyd

**Published:** 1869-07

**Authors:** 


### Editorial.

---

### NITROUS OXYD.

This agent still receives a good share of attention from the Dental profession. The subject is not yet well understood. After a constant and undivided devotion of mind and body to its consideration for a term of years, I feel my lack of knowledge in regard to it more than at the beginning of the investigation. Still I may be able to point out some mistakes into which other experimenters have fallen, and this, in all kindness, I will endeavor to do, to a limited extent.

Because protoxyd of nitrogen is rich in oxygen, and because it supports combustion, it has been claimed that, of course, it supports respiration by furnishing oxygen to the blood. Others claim that because the elements composing it are chemically united, it can not yield oxygen to the blood; and they illustrate their position by a reference to binoxyd of nitrogen, or nitric oxyd, which acts as a deadly poison when inhaled. Now, the theorizing is as bad in the one case as in the other, and in both is objectionable. The stereotyped phrase that in atmospheric air the nitrogen merely dilutes the oxygen, is as wide off the mark as either of the above theories, for the oxygen would occupy the same space it does if the nitrogen were obliterated.

Like all chemical substances, (and matter is all chemical) nitrous and nitric oxyd must be examined, each on its own merits.

In nitrous oxyd the elements are held together by a feeble affinity and are, therefore, easily separated in obedience to stronger affinities. On this principle it supports combustion, and on the same principle *may* support respiration. But we must not *infer* that it does so. Chemistry tolerates inferences when they are backed by demonstrations. There is some vagueness in reference to what is meant by supporting respiration. It is ordinarily used almost exclusively in connection with getting oxygen *into* the blood; but, in our waking hours, it comes much nearer meaning getting carbonic acid and water *out* of the blood.

It is true nitric oxyd is twice as rich in oxygen as nitrous oxyd; but it is not true that it is as ready to part with it. On the contrary, it most urgently demands more oxygen. So energetic is its affinity for oxygen that it takes two equivalents of this element from atmospheric air almost instantaneously, and the compound thus formed takes still another from water, if it be present. Nitric oxyd might be practically used as a deoxydizer much more extensively than it is. The terrible depression of vital force resulting from breathing nitric oxyd, even very much diluted, (for only thus it can be breathed) is partly and perhaps mainly due to its affinity for oxygen. Commingled with the blood, it takes the oxygen of reserve from the blood corpuscles, and they fail to support life in proportion to the degree of deprivation. But this is only half the story of its toxic effects when breathed. We have noticed that it rapidly takes two equivalents of oxygen from the atmosphere, and the result is nitrous acid. This in contact with watery vapor is changed to nitric acid, by its taking another equivalent of oxygen. This, with the increased energy due to its nascent condition, cauterizes the mucous membrane lining the air cells, and does any one suppose that when thus cauterized the membrane is in proper condition for the transmission of gases in either direction?

With nitrous oxyd the case is radically different. It is ever ready to *give* and but little inclined to *take* oxygen; and when inhaled *may* pass into the circulation and *may* yield oxygen to the blood. But does it? Just here let experience take the place of theory.

When pure nitrous oxyd is taken into the air cells it does not *all* come out again. This we have demonstrated scores of times. When respired there is usually a great increase of carbonic acid in the earlier expirations. This is beyond dispute. From breathing large doses of it the urine is increased in quantity, and contains more oxydized matter. These facts I have repeatedly demonstrated on myself and others. It will *sustain respiration a long time*, and it does so either by furnishing oxygen to the blood or by carrying off the carbonic acid and water, so that the oxygen of reserve can supply the vital functions, or it may act in both these ways, for they are not incompatible, as is constantly proved by atmospheric air performing both offices in respiration. I have many times seen nitrous oxyd breathed for twenty minutes, while atmospheric air was rigidly excluded. I have so breathed it many times myself, and without the slightest loss of consciousness, and what is more, without change of complexion, sense of suffocation, or any inconvenience whatever. The results were the same with the others who did so, except that there was more or less complete loss of consciousness. One time I breathed it exactly an hour, taking just eleven breaths of air during the experiment, and was sufficiently conscious and self-possessed to thrust a protected steel instrument into my thigh once a minute, as was corroborated by counting the holes in the skin after the close of the experiment. During the greater part of the hour the respirations were from three to seven to the minute, while the pulse, frequently counted, was not found below sixty-seven nor above seventy-two. At no time during the hour was there any darkening of the complexion or sense of suffocation. The after effects will be omitted here for want of time and space. The experiment is not a prudent one.

In view of the above facts, my venerable friend of Syracuse, and my younger one of St. Louis, will excuse me if I state that a person can "live longer in such an atmosphere than he could live under water," and that a person would *not* "die as speedily from the inhalation of nitrous oxyd alone, without the simultaneous respiration of atmospheric air, as he would to breathe any gas which was deprived of oxygen."

In conclusion, we are not disposed to claim any credit for being able to rescue our friends from the mistakes into which they have fallen; for, though we have probably spent a month in the investigation of this subject for each day spent thus by them, they were busily engaged in other parts of the field of science, finding out facts and principles which we wanted to know, and many of which they have kindly told us already.

Brethren, let us candidly, frankly and dispassionately investigate this subject. If we fail to do so, it is likely to be neglected, for the medical profession has not the time or lacks the inclination to attend to it. Besides, anæsthesia is our baby. Let us nourish, cherish and protect it. W.

